# Report of the Diseases of Edinburgh for January, 1810

**Published:** 1810-03

**Authors:** John Roberton


### *Report of the Diseases of Edinburgh for January*, 1810. By John Roberton, M. D.

For several days after the commencement of this month the weather was uncommonly pleasant. Indeed, it was so extremly mild that vegetation had, in a few days, made considerable progress. About the end of the first third of the month, however, the weather assumed a very different aspect. It became cold and disagreeable for two days; great quantities of snow had fallen on the neighbouring mountains, and a similar fall, about the middle of the month, took place in Edinburgh. In the space of a few hours, the snow fell fifteen inches thick, which is a much greater fall than we have had in this city for several years. For two or three days, almost immediately after, the weather became soft, and much of the snow was gradually dissolved; but, about the beginning of the last third of the month, the frost became very intense. For a day or two about this time, a circumstance took place which seldom occurs in this part of the world. A very thick fog prevailed all over the city, almost similar to the very dense ones which sometimes occur in London. Our fogs are almost always during soft weather, but in the present instance, the thermometer stood several degrees below the freezing point. The weather succeeding this, gradually became soft, and the snow was almost entirely dissolved in a few days. This was succeeded by a day or two of mild weather, and especially toward evening and in the mornings, fogs of considerable density overspread the city and vicinity. The remaining days of the month were extremely mild.

The barometer underwent but little variation during the whole month: it was rather high about the beginning of it, and was much in the same situation at its termination.

The thermometer, about the beginning of the month, was high, but, during the succeeding frost, it was often several degrees below the freezing point. During the thaw that succeeded, it again rose, and continued between 36 and 40 till the termination of the month.

The diseases which, during this period, have caused the greatest mortality, were fevers, small-pox, pneumonia, and enteritis. The last of these, however, did not so often prove mortal, during its active state, as afterward happened from dropsical effusions in consequence of the previous inflammation. There were, besides these now enumerated, a very great variety of complaints of a more trifling nature, which were not dangerous, but only proved troublesome during their existence. Such as catarrh, cynanche tonsillaris, rheumatism, and slight, but general eruptions on the skin.

The fevers, which, as usual, are of the typhoid kind, have, since last month, been greatly aggravated in their malignity; they have consequently proved mortal in various confined and dirty parts of the City.

The small-pox continue much the same as last month, and a great many of the people here not only now disbelieve the efficacy of cow-pox inoculation, but roundly assert that those who are subjected to it, are afterward affected with many other diseases, which, under other treatment, they would have escaped. To encounter the stubborn prejudices of an ignorant mob, is too hard a task for one person; I must therefore only lament, with all friends to important improvements, that such an evidently beneficial discovery should admit of doubt, or have its utility misrepresented.

The inflammatory diseases still continue milder than in former former seasons. Some severe cases, however, of pneumonia, are occasionally to be met with in practice. Enteritis too, of considerable severity, sometimes occurs. Various instances of this disease terminating in dropsy, have, especially in patients advanced in life, been met with.

The cases of catarrh, cynanche tonsillaris, and rheumatism, have been very numerous. The first of these has yielded to the repeated administration of purgative medicines, the second to them, with the frequent use of gargles, and rheumatism to sudorific medicines, and when the acuteness of the symptoms have abated, to the application of friction. In all of these last mentioned complaints, preserving the body in an equal and dry temperature was indispensibly necessary.

The eruptions in the skin, though only troublesome during their existence, have been very general. In almost every case, a solution of muriate of mercury in spirit of wine has removed them in a few days.

